# Supplementary material for: Patterns of Intron Gain and Loss in Fungi
Source: PLoS Biol. 2004 Nov 30;2(12):e422. doi: 10.1371/journal.pbio.0020422 (PMC532390; doi:10.1371/journal.pbio.0020422)
Supplement: Table S1 — Also available at http://genes.mit.edu/NielsenEtAl/. (4.3 MB ZIP). [file pbio.0020422.st001.zip › NielsenEtAl/html/347.html]

AN0896.1.NCU03031.1.MG00666.1.FG00743.1


```
 CLUSTAL W (1.82) Multiple Sequence Alignments - Introns Inserted


Sequence 1: NCU03031.1	166 aa
Sequence 2: FG00743.1	197 aa
Sequence 3: MG00666.1	171 aa
Sequence 4: AN0896.1	171 aa
Alignment Length: 201 aa
Number Identitical Residues: 70 aa
Alignment Score (without introns) 3869


MG00666.1 	MASIARP--LRQVACRAG---PTSLAQRTLANNVPR-----IAAFHASSRRN-LLPPLPQ
NCU03031.1	MASVARSSALLK----------QVAAQQSVAANGLR-----VAAFHTTSRKS-LLPPPPQ
FG00743.1 	MASIARSSLLRQTAMASRLATAAIPATRSTSMPSIRTQLKGVAAFHNTTRRSAILPPGPQ
AN0896.1  	MASLMRQSCLSAFR--------APLATRNTVG-VSQ-----VVAFHASAKKQ-ILPPLPQ
          	***: * : *               * :.      :     :.*** ::::. :*** **

MG00666.1 	1---KIQGT-~---V1NDPAPVPSPSPSHGHYHWTFER~ILAAGLVPLTVAPFAAGSFNP
NCU03031.1	1---RIEGT-~---V1NDPVEVPPPSPSHGSYHWTFDR~VVAAGLIPLTVAPFAAGSLNP
FG00743.1 	1PVERIEGG-~---I1NEPAPVPEPSSTHGSYHWTFER~FLAAGLVPLTVAPFAAGSLNP
AN0896.1  	1---TIQGTT1FCAV~NDPAPIPKPHPSHGSYHWTFER2LVAVGLIPLTIAPFAAGSLNP
          	     *:* :  .:: *:*. :* * .:** *****:* .:*.**:***:*******:**

MG00666.1 	VMDAVLCSTLLLHSHLGLQ~NVITDYLPQKRVPKSRKAAMWLLNLATAVVGVGLYEFETN
NCU03031.1	TMDAVLAATILIHSHTGFG~NIIVDYVPSKRVPKARKVFTWGLNAATVLVGLALYEFETT
FG00743.1 	TLDAILCSVLLLHSHMGFQ~QVVIDYIPSRTYPGLRKTFNWLLNIATVLVGVGLYEFETN
AN0896.1  	VTDAVLCSLLVLHSHVGFQ2ASIIDYFPTRRVPKTSTLLNWLLRGFTLTTAVGLYELETN
          	. **:*.: :::*** *:    : **.* :  *   .   * *.  *  ..:.***:**.

MG00666.1 	DVGLTEGVRRIWTA~------------
NCU03031.1	DVGLTETIKRVWKA~------------
FG00743.1 	DVGITEAVRRVWKA2FMTAGICIENFL
AN0896.1  	DVGLTETIKRVWKA~------------
          	***:** ::*:*.*
```
